# A Unique Presentation of a Giant Fibroadenoma in a Perimenopausal Female

**DOI:** 10.7759/cureus.60189

**Published:** 2024-05-13

**Authors:** Abhilasha Bhargava, Imran Ali Khan, Darshana Tote, Anup Zade, Yogesh B Manek

**Affiliations:** 1 General Surgery, Jawaharlal Nehru Medical College, Datta Meghe Institute of Higher Education and Research, Wardha, IND; 2 General Surgery, Mahatma Gandhi Institute of Medical Sciences, Wardha, IND

**Keywords:** middle-aged women, phyllodes tumor, mastectomy, giant fibroadenoma, breast reconstruction

## Abstract

Giant fibroadenomas are common in young females and are rarely reported in perimenopausal or menopausal females. These fibroadenomas are observed as single, mobile, small to large, with distinct boundaries. These tumors are hyperplastic and characterized by their aberrant growth in both the epidermal and mesenchymal layers, which can be accompanied by pain in some instances. These tumors have similar clinical resemblances to other epithelial and stromal tumors, such as phyllodes tumors, except for the level of disease severity and malignancy. Treatment of giant fibroadenomas includes surgical resection. Surgical excision is done by complete excision of the fibroadenoma, with the rest of the breast tissue and the nipple-areolar complex preserved. Timely diagnosis can be helpful in the prevention of adverse outcomes. This is a case of a 40-year-old female who presented with a lump in her right breast, for which she underwent a wide local excision. On histopathology, it was found to be a giant fibroadenoma. Her postoperative recovery was uneventful.

## Introduction

Fibroadenomas of the breast are benign tumors usually of solitary, hard, and rubbery consistency with slow progression [1]. Fibroadenomas that are typically greater than 5 cm in size or weigh more than 200-500 g are termed "giant fibroadenomas" and are commonly observed in young adults [1-3]. It has been found to affect females between 20 and 30 years of age [3]. Giant fibroadenomas are noted to consist of 0.5%-2.0% of the total cases of fibroadenomas. Giant fibroadenomas are difficult to diagnose and manage as they can have a similar clinical presentation as other breast tumors, such as phyllodes, in terms of the involvement of epithelium and stromal cells [2,4,5]. Giant fibroadenomas are sometimes observed as mobile, palpable masses with no definite boundaries and texture differences. These tumors pose a diagnostic challenge due to their rare incidence and unpredicted behavior [3,4]. Normal physical examination and standard radiological examinations such as ultrasound and mammograms might not be enough to conclude the diagnosis; additional histopathological analysis can be helpful in the final diagnosis [6]. We present a case of a 40-year-old female with an asymmetric right breast due to a lump that was insidious in onset and progressive in nature, which offered a diagnostic dilemma.

## Case presentation

**Figure 1:**
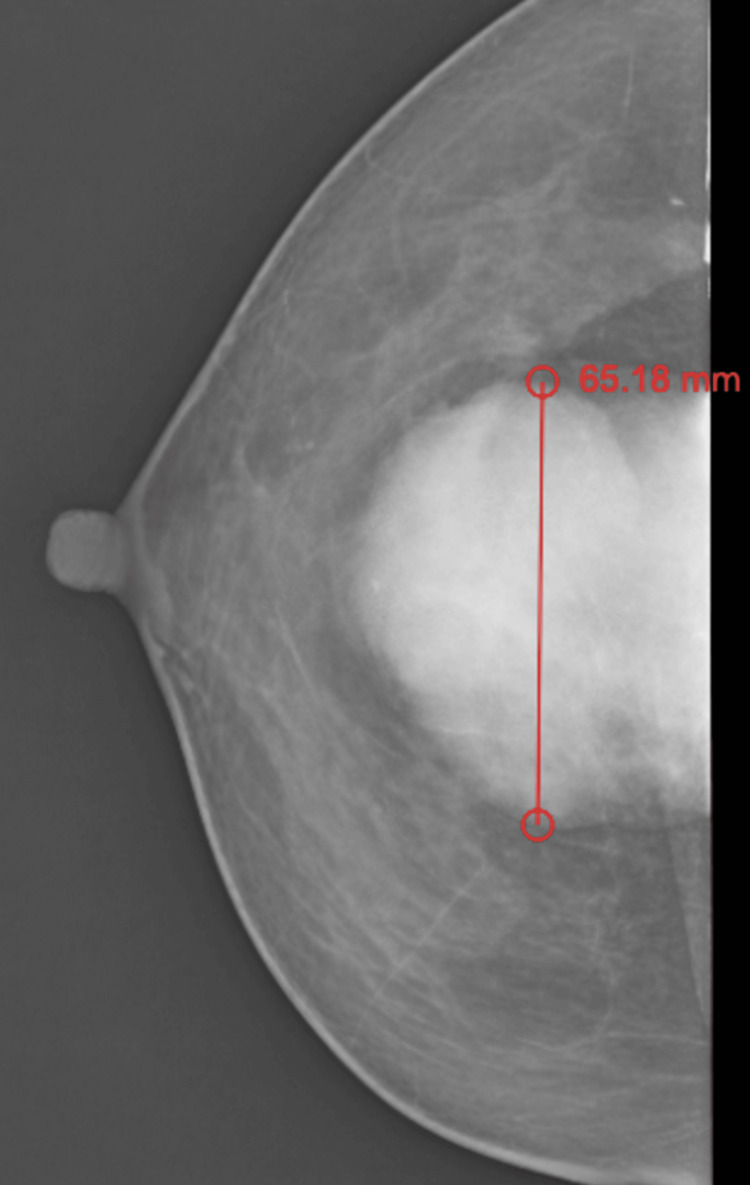
Mammography of the patient depicting the presence of a giant fibroadenoma

This is a case report of a 40-year-old female who visited our hospital with a lump in the right breast. She noticed the lump one year ago, which was insidious in onset and gradually progressed to its current size of 6 x 3 cm. The lump presented with pain over the right breast, which aggravated during her menstrual cycle. There was no evidence of any ulceration, fungation, retraction, dimpling, or discharge from the breast. She neither had any significant past medical history nor a family history of any breast pathology. She had regular menstruation cycles and was not on any hormonal medications. Physical examination of the right breast revealed a well-defined, spherical mass of 6 x 3 cm, which was mobile and firm in consistency, in the retroareolar region involving the upper and lower quadrants of the breast. The skin over the swelling and nipple-areolar complex was insignificant. There was no evidence of any axillary lymphadenopathy. All the routine blood investigations were carried out, and they were found to be within normal limits. The mammography report revealed a well-defined radio-opaque mass of 6.4 x 6.3 x 4.3 cm with spiculated margins in the outer lower quadrant of the right breast, 3.7 cm away from the nipple-areolar complex, which was reported as BI-RADS category 4 (Figure 1).

**Figure 2:**
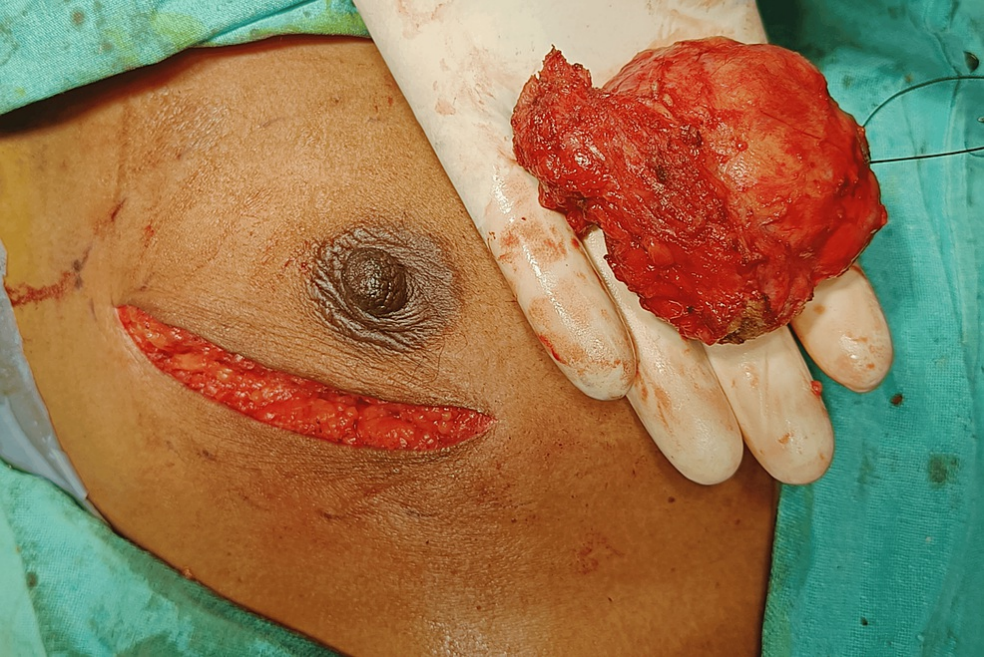
Intraoperative image showing the incision and excised sample of the giant fibroadenoma

A tru-cut biopsy was performed on the lump in the right breast. The biopsy results indicate the presence of neoplastic breast gland tissue surrounded by myxoid stroma and a part of the capsule. These findings suggest the presence of fibroadenoma in the breast. No malignant cells were observed. The patient was further subjected to excision of the fibroadenoma. Due to the large size and spiculated margins of the lump on mammography, the incision was planned in a way that it could be included in the modified radical mastectomy incision if it were required at a later stage after the histopathology report (Figure 2).

**Figure 3:**
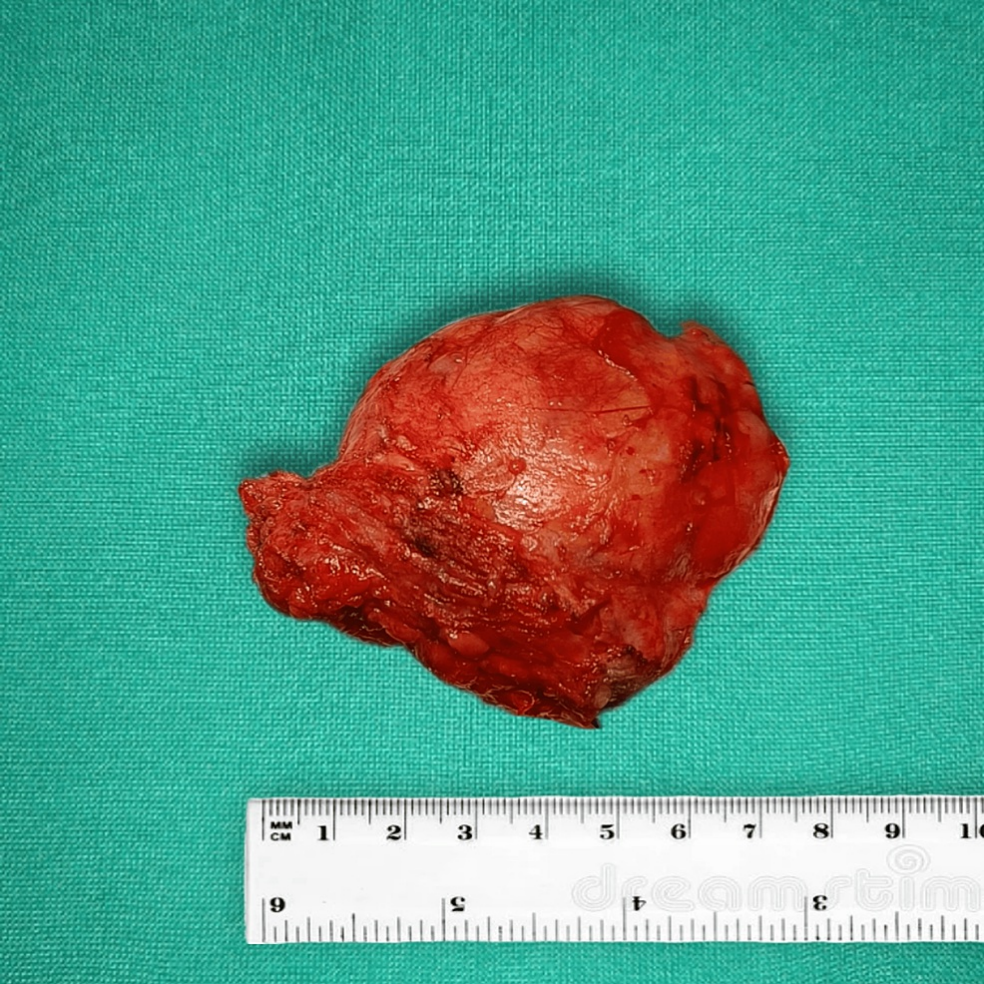
Excised sample of the giant fibroadenoma

The excised specimen was sent for histopathological analysis (Figure 3).

**Figure 4:**
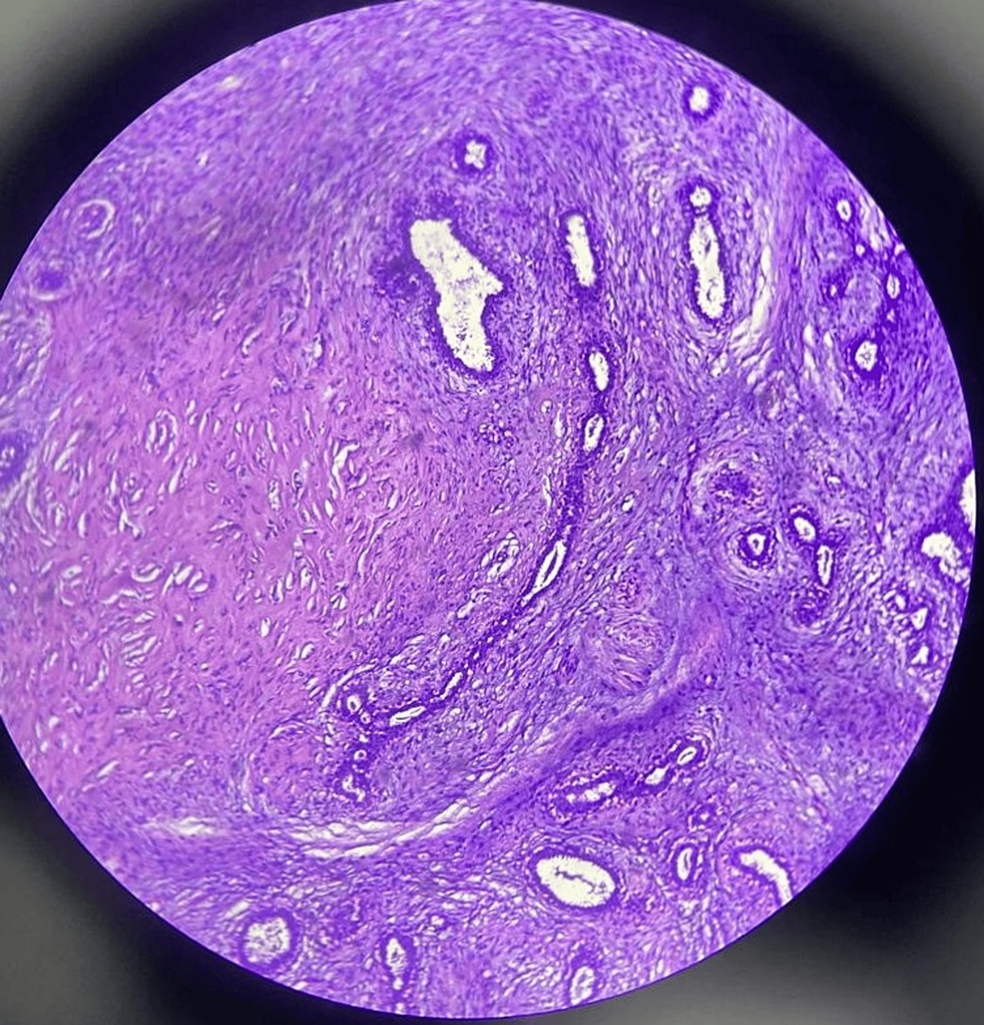
Histopathology slide of the giant fibroadenoma

The histopathology report of the excised specimen of 7 x 7 x 3 cm showed myxoid changes, which confirmed the diagnosis of giant fibroadenoma (Figure 4).

**Figure 5:**
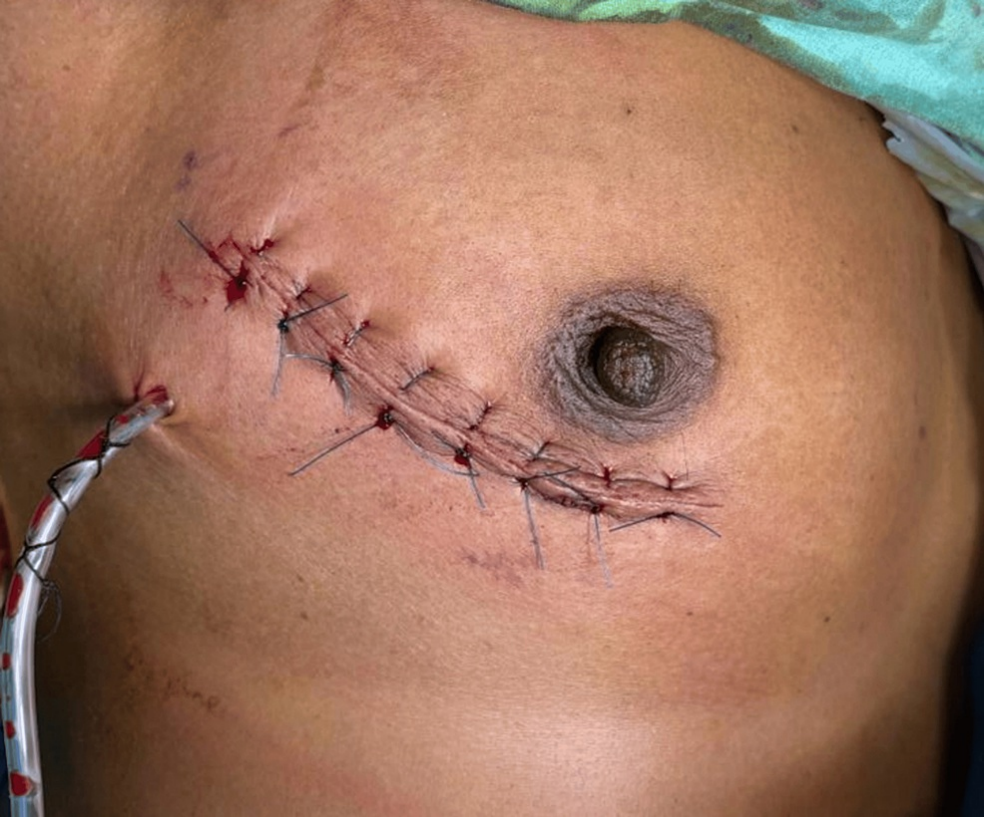
Postoperative image of the patient

The procedure was uneventful, with no postoperative complications. Postoperative day four examination showed a healthy suture line with no evidence of discharge and a good recovery (Figure 5).

The patient was discharged on postoperative day seven. The two-month follow-up showed a healthy scar line with no fresh complaints.

## Discussion

Giant fibroadenomas are not clearly understood due to their rare incidence and uncertain clinical behavior. This poses a diagnostic and management challenge [5]. Giant fibroadenomas are reported to be similar in clinical presentation and pathophysiology to phyllodes tumors and virginal hypertrophy [7]. These tumors are commonly reported to affect a single breast, though there are reports of bilateral breasts being affected as well [3,8]. Routine radiological diagnostic tools are less recommended due to their lower efficiency in concluding the final diagnosis of fibroadenomas. MRI and computed tomography can be used as additional screening modalities, as mentioned in the published research literature [6,9]. Treatment of fibroadenomas includes local surgical excision, though it has been associated with recurrence [3,7,10]. Due to their massive size, giant fibroadenomas of the breast essentially need surgical resection as they may affect the physical and psychological condition of the patient. Additionally, these tumors impose a high physiological risk in the form of vessel congestion, distortion of the glands, necrosis, and, in some cases, ulceration of the breast [6,9]. The preoperative histopathology carried out by tru-cut biopsy confirmed the diagnosis of fibroadenoma, with major growth of the tumor in the past year, which was also the prime concern of the patient. Hence, she was advised surgical excision of the fibroadenoma, which was similar to the case reported by Nwashilli and Obahiagbon [1]. The patient underwent wide local excision of fibroadenoma for giant fibroadenoma with a good outcome.

## Conclusions

Giant fibroadenomas are large tumors with a polyclonal proliferative nature, easy access to the nodules, and are rarely metastatic. Better outcomes with the preservation of the breast tissues and the nipple-areolar complex can be expected with timely diagnosis and treatment. Surgical excision of the giant fibroadenoma was performed, as in this case, with regular follow-ups to monitor recurrence.
